# O.5.3-1 Implementation of an online group model for work-related well-being among sport instructors

**DOI:** 10.1093/eurpub/ckad133.252

**Published:** 2023-09-11

**Authors:** Mari Punna, Tiina Laiho, Sirpa Laitinen-Väänänen, Paula Harmokivi-Saloranta

**Affiliations:** School of Professional Teacher Education JAMK, Finland; Haaga-Helia University of Applied Sciences, Finland; mari.punna@jamk.fi; School of Professional Teacher Education JAMK, Finland; Haaga-Helia University of Applied Sciences, Finland; mari.punna@jamk.fi

## Abstract

**Purpose:**

Sports instructors, who work in small and mid-sized municipalities and the public sector, provide physical activity counselling and group guidance for municipality residents of all age groups. Sport instructors execute a variety of physical activity and well-being promotion activities. Many of them are working independently, having only little collegial or community support. Additionally, only little is known about work-related well-being of their own as well as methods on how to improve it.

**Project description:**

Within research and development project *TOGETHER -Towards an online community among sport instructors,* an online group for work-related well-being was created and piloted. There were 32 participants who were working as sport instructor in small or mid-size municipalities in Finland. Before the start of the group meeting process, the participants were interviewed about their perceptions about work-related well-being. The group process consisted of six online small group meetings with four to six participants and the facilitator in a group. Each of the biweekly group meetings took 1,5 hours. The themes of group meetings were pre-stated, nevertheless, the participants' own reflections and discussions were highlighted. The main themes focusing on work-related well-being were sense of community, autonomy, capability and social support.

The participants were instructed to set their own aims for their individual work-related well-being process. Between the group meetings the participants have made written reflections about the themes and the process. After the last group meeting, group interviews will be made to find out the outcomes and reflections of the process. They will be shown in the presentation.

**Conclusions:**

Based on the reflections and findings of the implemented online group meeting process, the model will be further modified. The final outcome of the project will be a tested online model for increasing work-related well-being among sport instructors working in municipalities, which will be presented.

The research has been funded by the Finnish Work Environment Fund.

